# Supplementary material for: Identification of an H-Ras nanocluster disrupting peptide
Source: Commun Biol. 2024 Jul 9;7:837. doi: 10.1038/s42003-024-06523-9 (PMC11233548; doi:10.1038/s42003-024-06523-9)
Supplement: Supplementary file 2 — Supplementary Information [file 42003_2024_6523_MOESM2_ESM.pdf]

Supplementary Information

Identification of an H-Ras nanocluster disrupting peptide

Candy Laura Steffen [1,*], Ganesh babu Manoharan [1,*], Karolina Pavic [1], Alejandro Yeste-Vázquez [2,3], Matias Knuuttila [4], Neha Arora [5], Yong Zhou [5], Harri Härmä [6], Anthoula Gaigneaux [7], Tom N. Grossmann [2,3], Daniel Kwaku Abankwa [1,4]

[1] Cancer Cell Biology and Drug Discovery group, Department of Life Sciences and Medicine, University of Luxembourg, 4362 Esch-sur-Alzette, Luxembourg.

[2] Department of Chemistry and Pharmaceutical Sciences, VU University Amsterdam, Amsterdam, The Netherlands.

[3] Amsterdam Institute of Molecular and Life Sciences (AIMMS), VU University Amsterdam, Amsterdam, The Netherlands.

[4] Turku Bioscience Centre, University of Turku and Åbo Akademi University, 20520 Turku, Finland.

[5] Department of Integrative Biology and Pharmacology, McGovern Medical School, UT Health, Houston, TX 77030, USA.

[6] Chemistry of Drug Development, Department of Chemistry, University of Turku, 20500 Turku, Finland.

[7] Bioinformatics Core, Department of Life Sciences and Medicine, University of Luxembourg, 4367 Esch-sur-Alzette, Luxembourg

[*] These authors contributed equally

Correspondence: daniel.abankwa@uni.lu

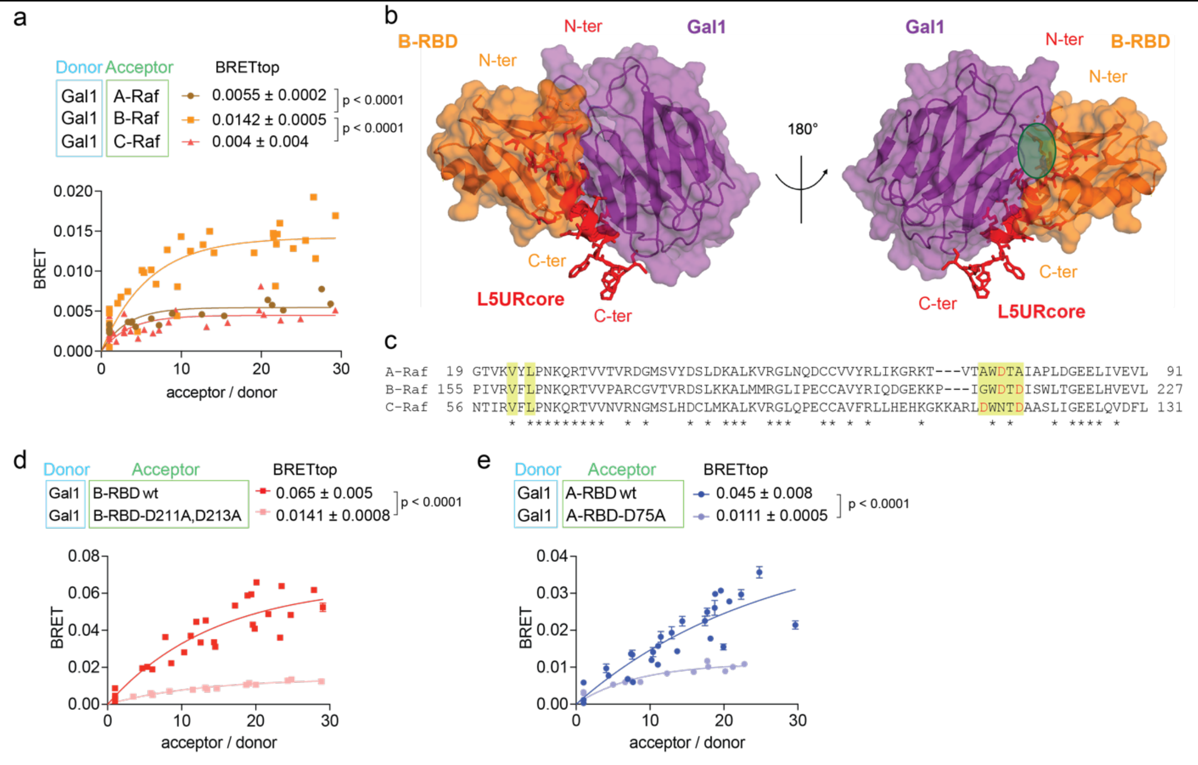

Supplementary Figure 1. Related to main Figure 1.

(**a**) BRET-titration curves of Gal1 and full-length Raf proteins; n = 3.

(**b**) Computational model of hypothetical Gal1/ B-RBD/ L5UR (22-45) complex indicating the carbohydrate binding site of Gal1 (PDB ID 3W58) in green. The structural model was created with PyMOL Molecular Graphics System (Version 2.5.1) using the published Gal1/ C-RBD docking model [1], the Haddock model of Gal1/ L5UR(22-45) [2] and the RBD structure of B-Raf (PDB ID 3NY5).

(**c**) Multiple sequence alignment of RBDs of A-, B- and C-Raf. The protein sequences of RBDs from the three human Raf proteins, A-Raf (P10398), B-Raf (P15056) and C-Raf (P04049) were essentially as employed in the cellular assays; in brackets Uniprot database (http://uniprot.org/) accession numbers. Multiple sequence alignment was performed using Clustal Omega (https://www.ebi.ac.uk/Tools/msa/clustalo/). Yellow highlighted residues were identified as possible interaction sites with Gal1 before [1], and mutations tested in the BRET experiments in (d, e) are in red.

(**d, e**) BRET-titration curves of Gal1 with wild-type (wt) A-RBD and A-RBD-D75A mutant (d); n = 3, or with wt B-RBD and B-RBD-D211A, D213A mutant (e); n = 3.

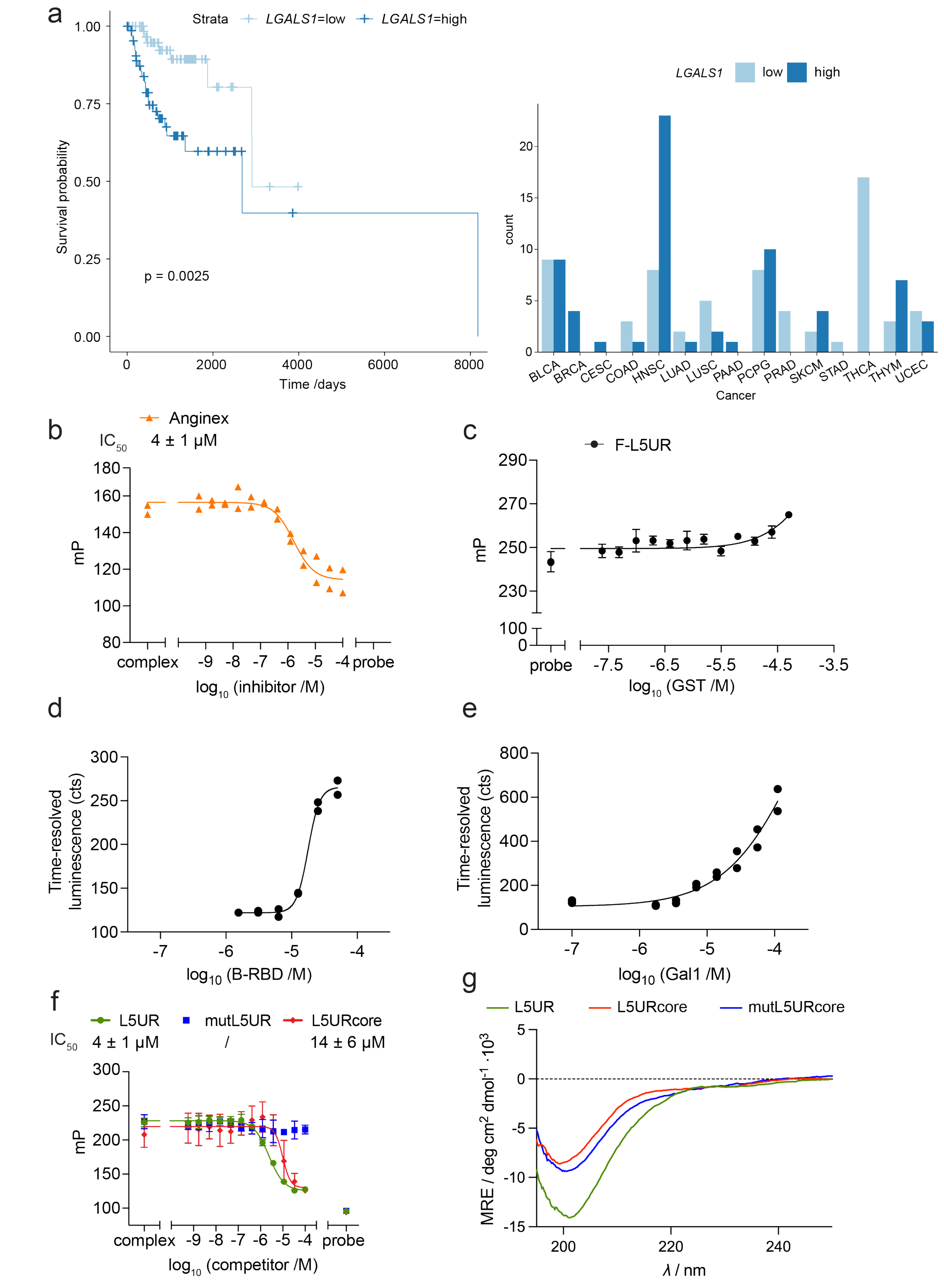

Supplementary Figure 2. Related to main Figures 2 and 3.
(**a**) PanCanAtlas data analysis reveals that high Gal1 (gene *LGALS1*) levels significantly decrease survival in HRAS mutant cancer cases (left). Higher Gal1 levels are more often found in head and neck (HNSC) cancers and to some extent in skin (SKCM) and thymus (THYM) cancers. These cancer types could therefore be particularly interesting for

treatment with a Gal1/ Raf-interface inhibitor, which would abrogate the stimulating effect of Gal1 on oncogenic H-Ras nanoclustering and thus MAPK-signalling.
(**b**) Displacement of F-L5UR (5 nM) from Gal1 (5 μM) by Anginex; n = 2.
(**c**) Control showing negligible binding of 10 nM F-L5UR to GST measured by fluorescence polarization; n = 3.
(**d**) Eu-L5URcore (29 nM) binding to B-RBD measured in the QRET assay using time-resolved luminescence detection; n = 2.
(**e**) Eu-L5URcore (29 nM) binding to Gal1 measured in the QRET assay using time-resolved luminescence detection; n = 2.
(**f**) Displacement of F-L5UR (5 nM) from C-RBD (200 nM) by L5UR-derived peptides; n = 3.
(**g**) Circular dichroism spectra of 25 μM of indicated L5UR-derived peptides in 1x PBS (pH 7.5).

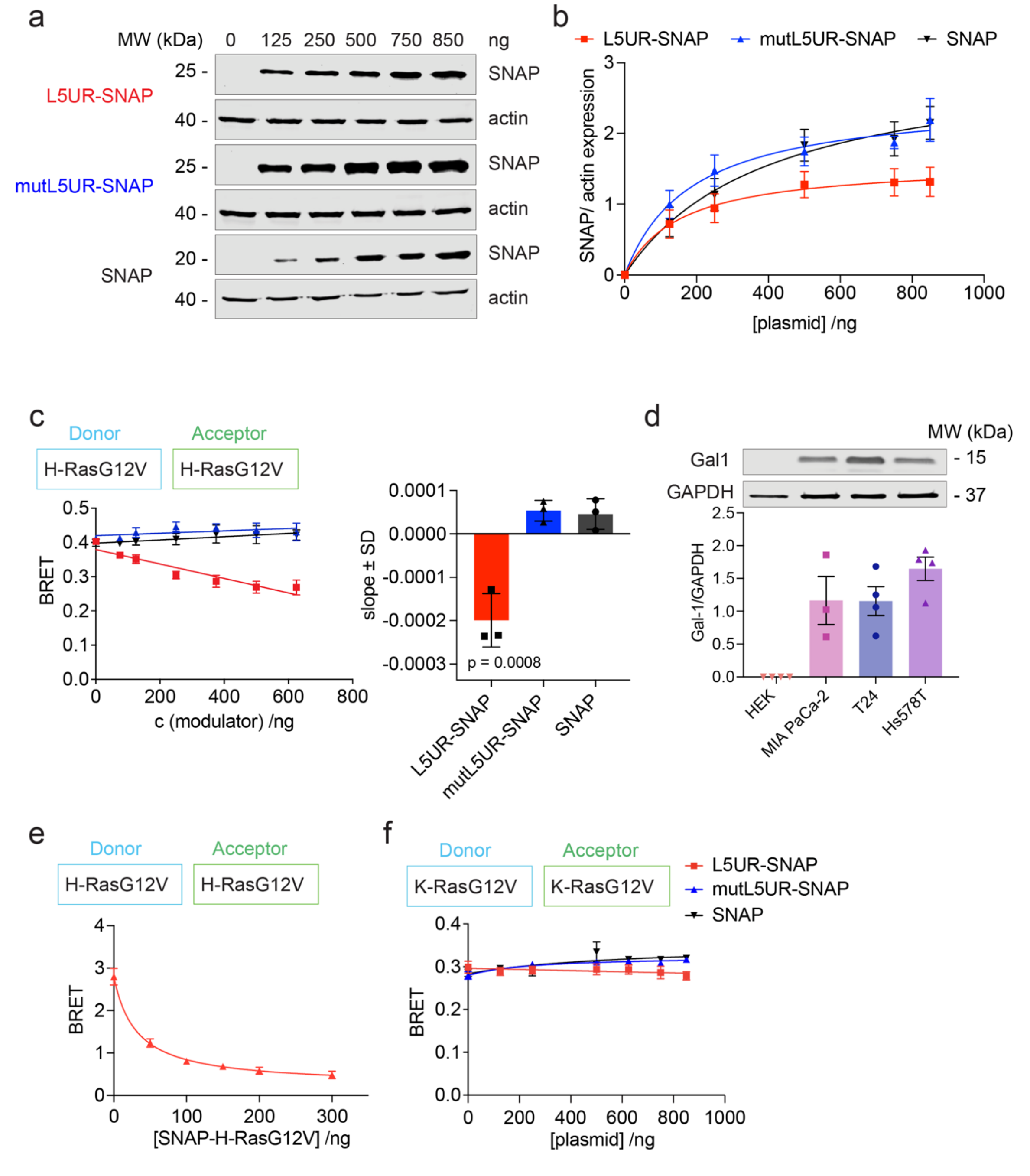

Supplementary Figure 3. Related to main Figures 3, 4 and 5.
(**a, b**) Representative immunoblots (c) and quantification of all repeats (d) showing dose-dependent expression of L5UR constructs (48 h); n = 3.
(**c**) Effect of L5UR construct expression (48 h) on H-RasG12V nanoclustering-BRET (donor:acceptor plasmid ratio = 1:5); n = 3.
(**d**) Immunoblot data and quantification of endogenous Gal1 expression in employed cell lines; n = 3.
(**e**) Effect of SNAP-H-RasG12V on NanoLuc/ mNeonGreen-H-RasG12V nanoclustering-BRET (donor:acceptor plasmid ratio = 1:5); n = 3.
(**f**) Negligible effect of L5UR construct expression (48 h) on K-RasG12V nanoclustering-BRET (donor:acceptor plasmid ratio = 1:10); n = 3.

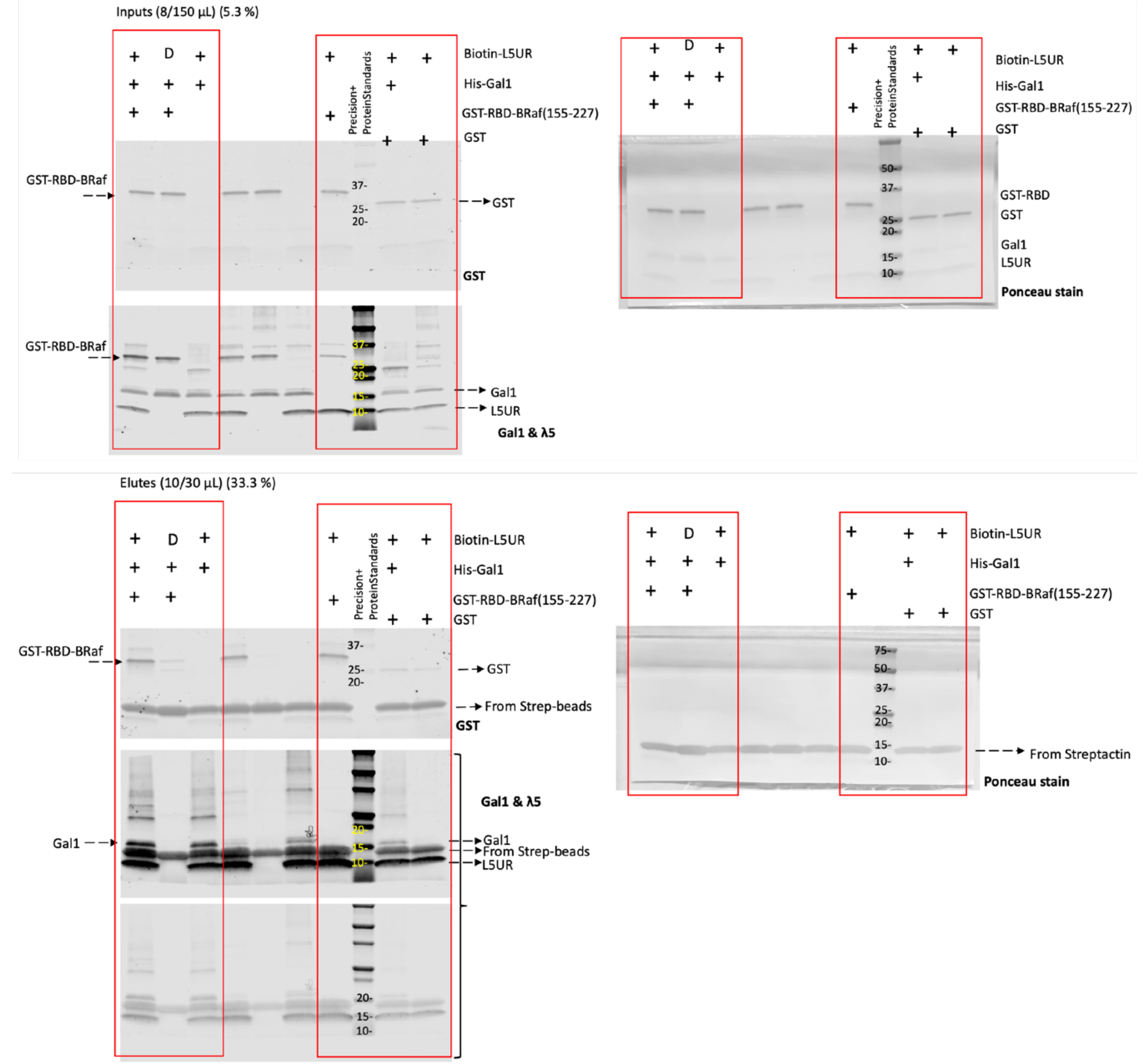

Supplementary Figure 4. Original Western blotting data of Figure 2c, repeat 1.

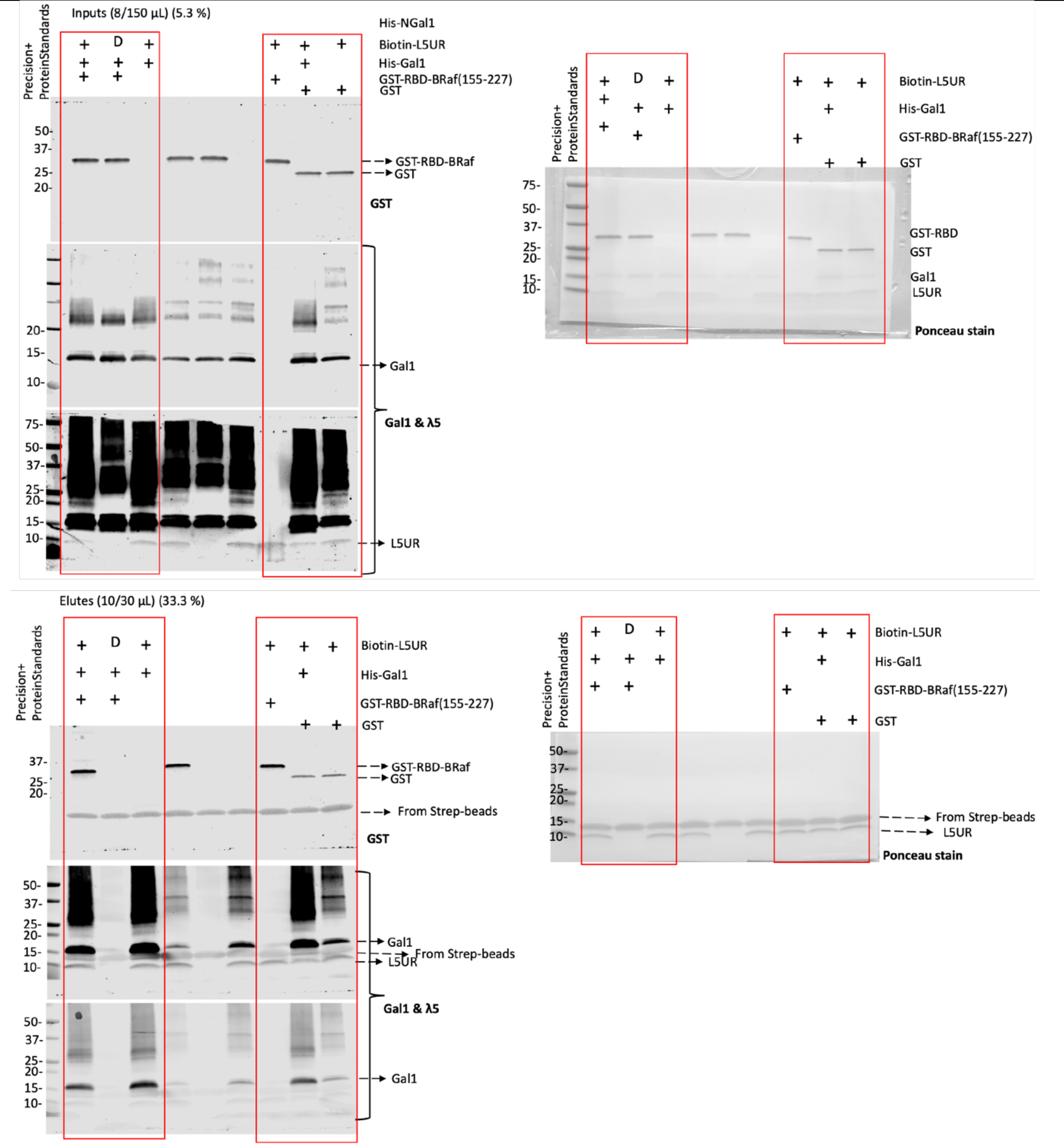

Supplementary Figure 5. Original Western blotting data of Figure 2c, repeat 2.

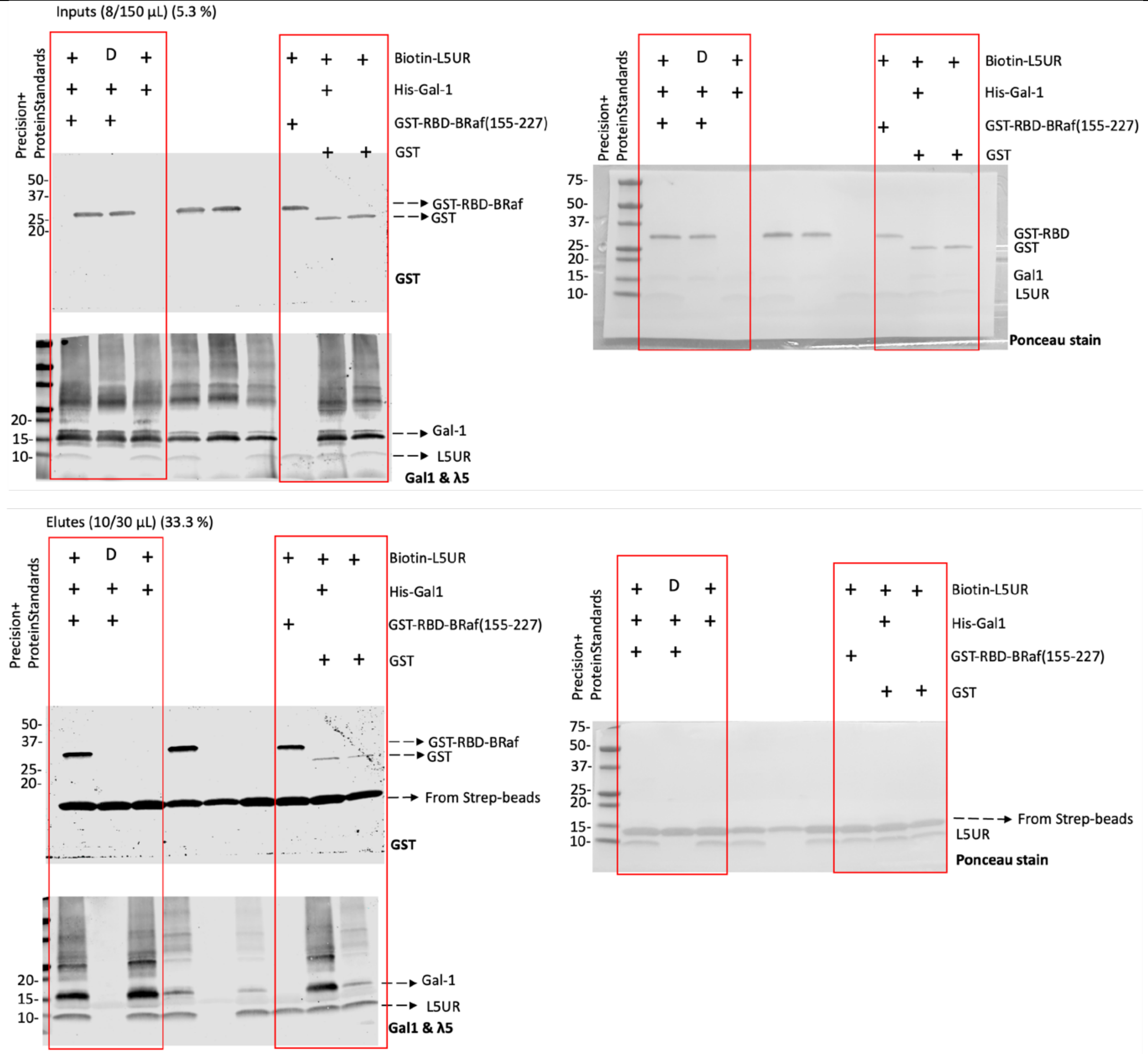

Supplementary Figure 6. Original Western blotting data of Figure 2c repeat 3.

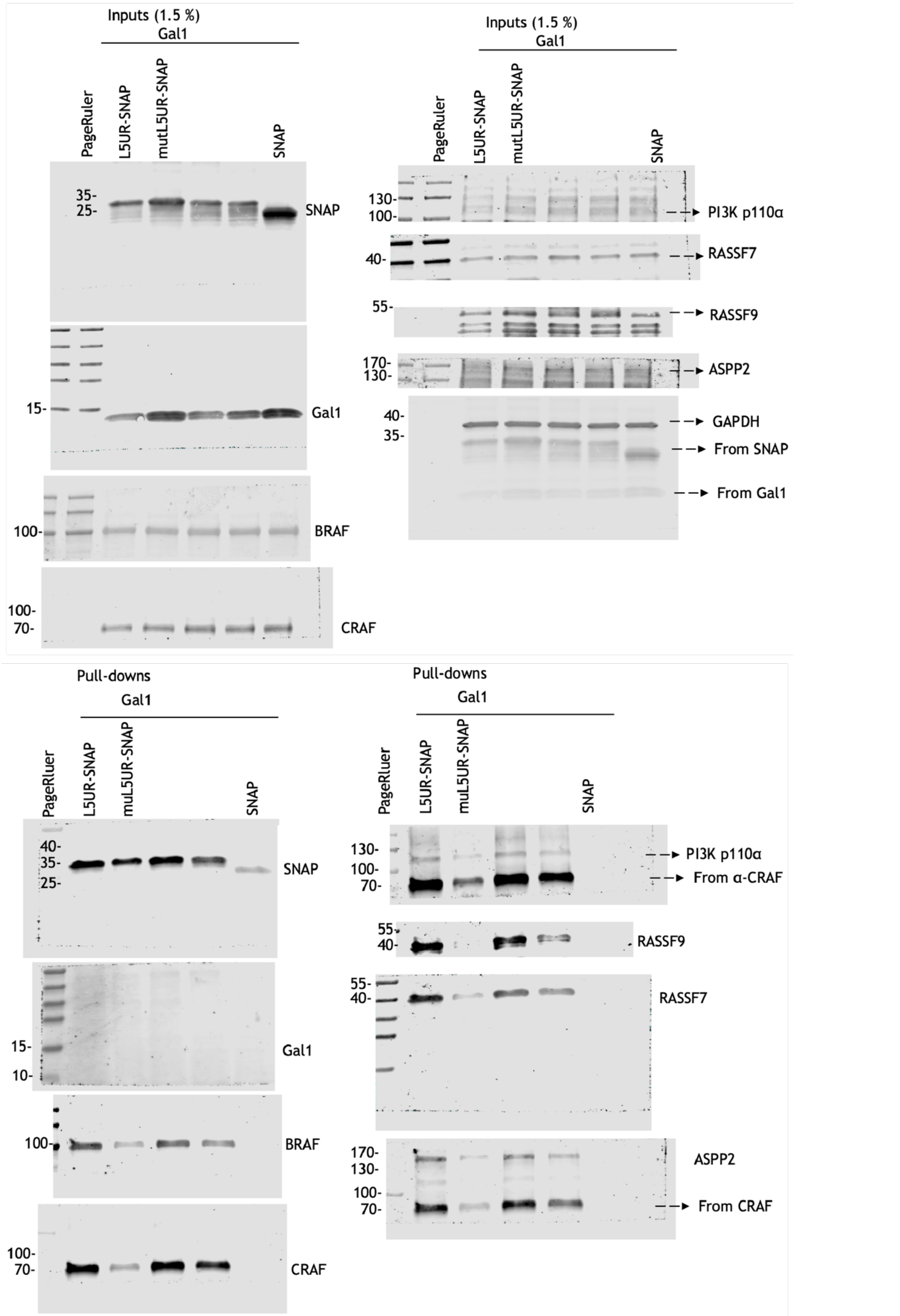

Supplementary Figure 7. Original Western blotting data of Figure 3e.

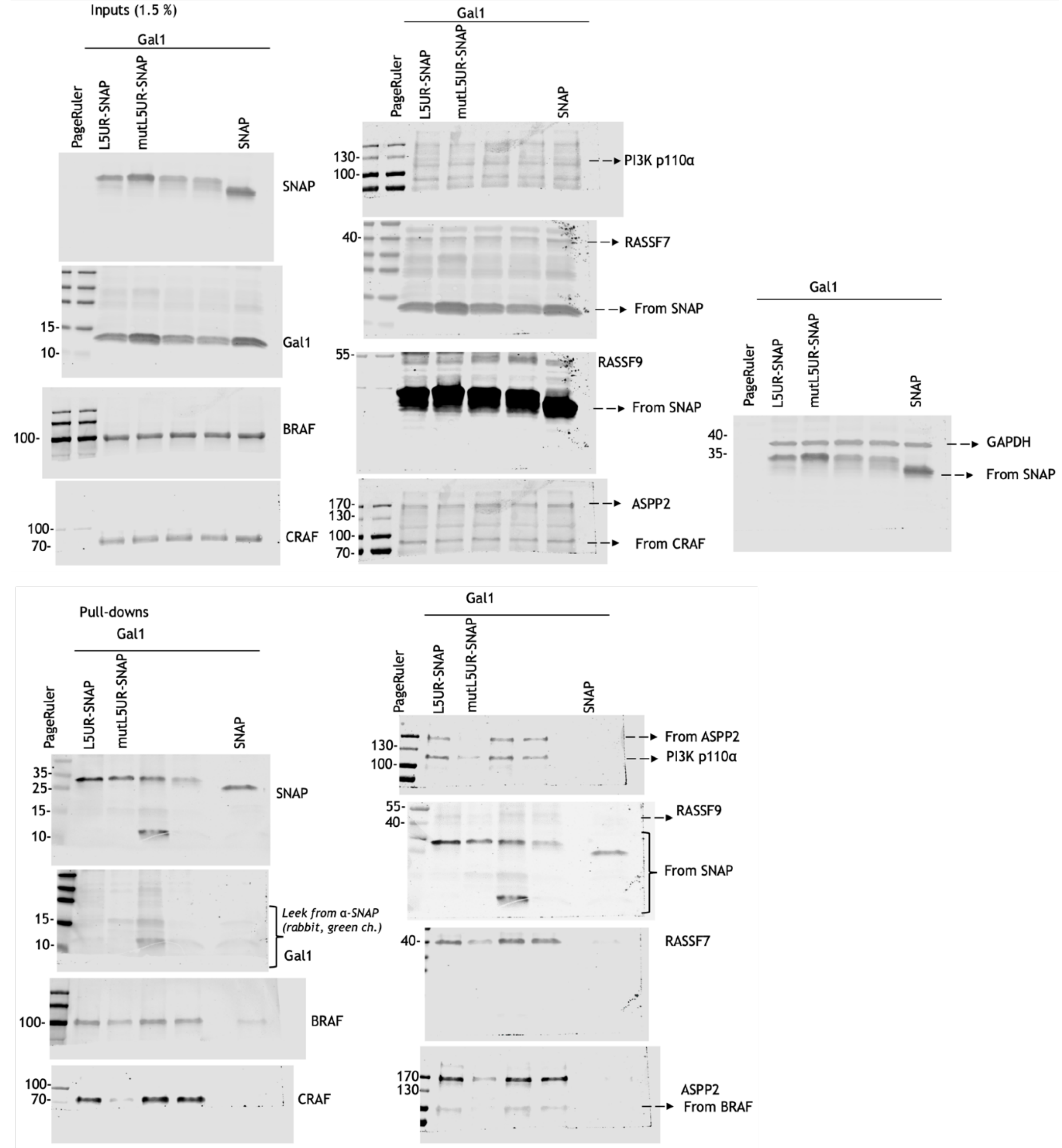

Supplementary Figure 8. Original Western blotting data of Figure 3e.

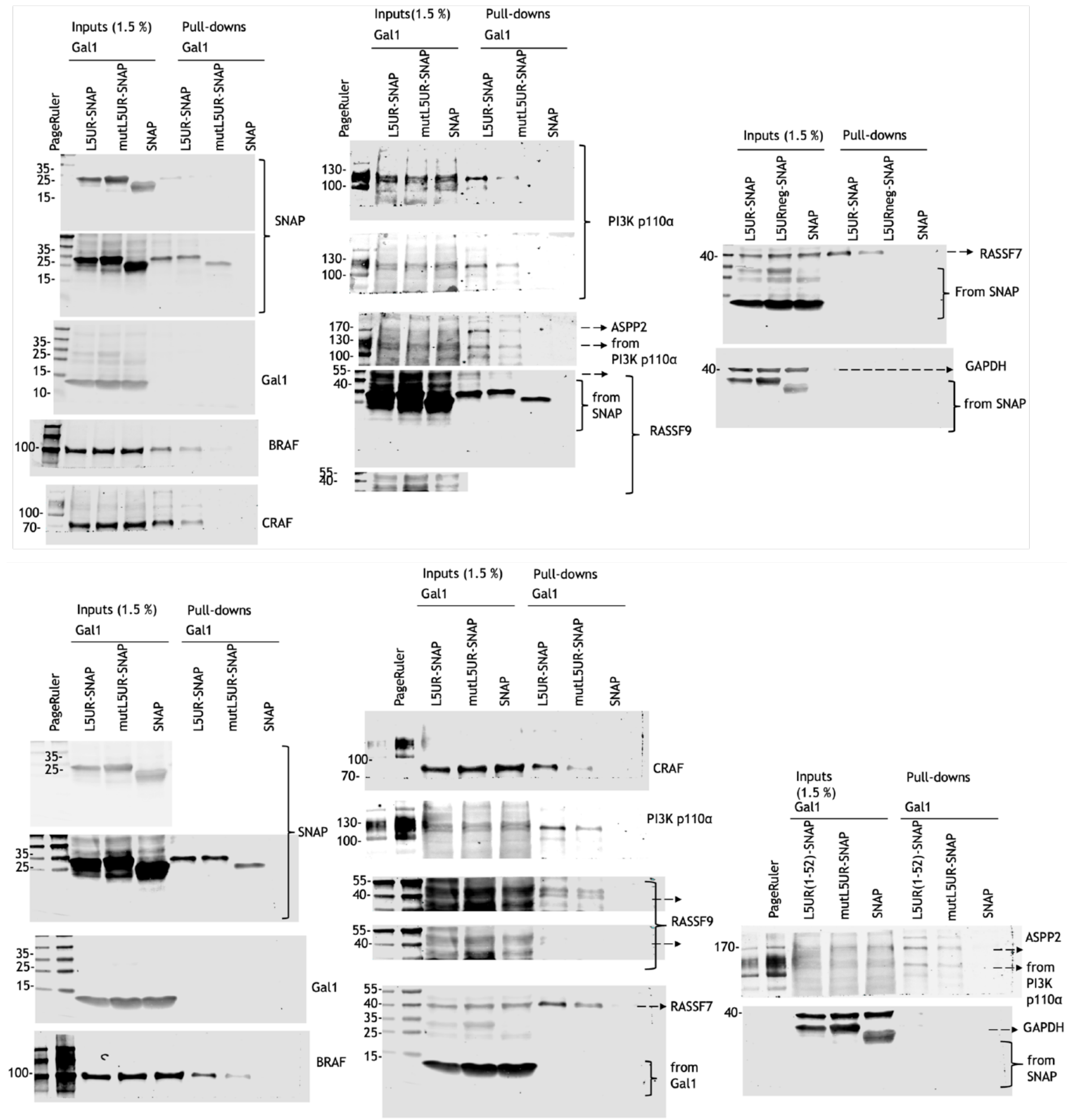

Supplementary Figure 9. Original Western blotting data of Figure 3e.

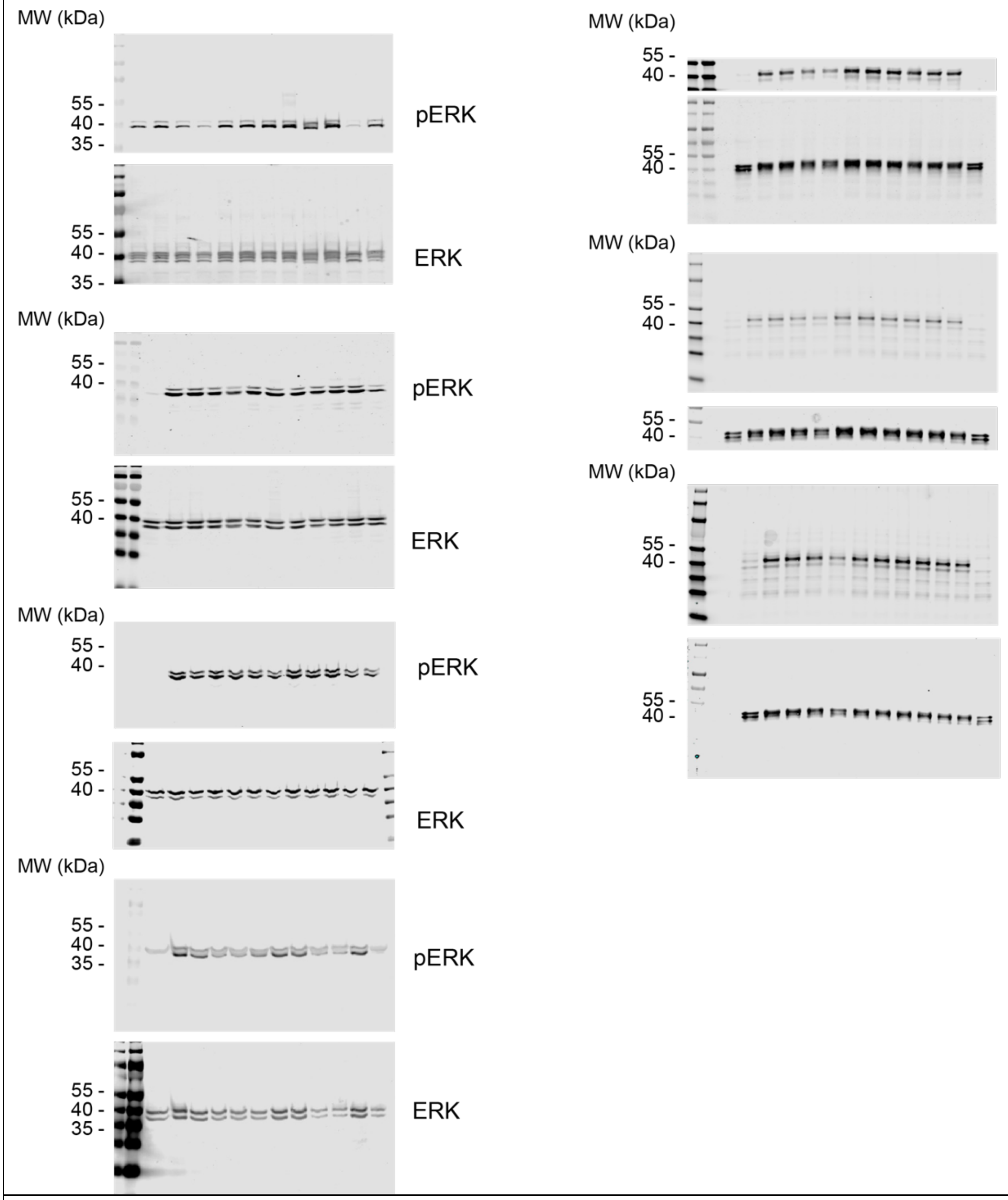

Supplementary Figure 10. Original Western blotting data of Figure 5a.

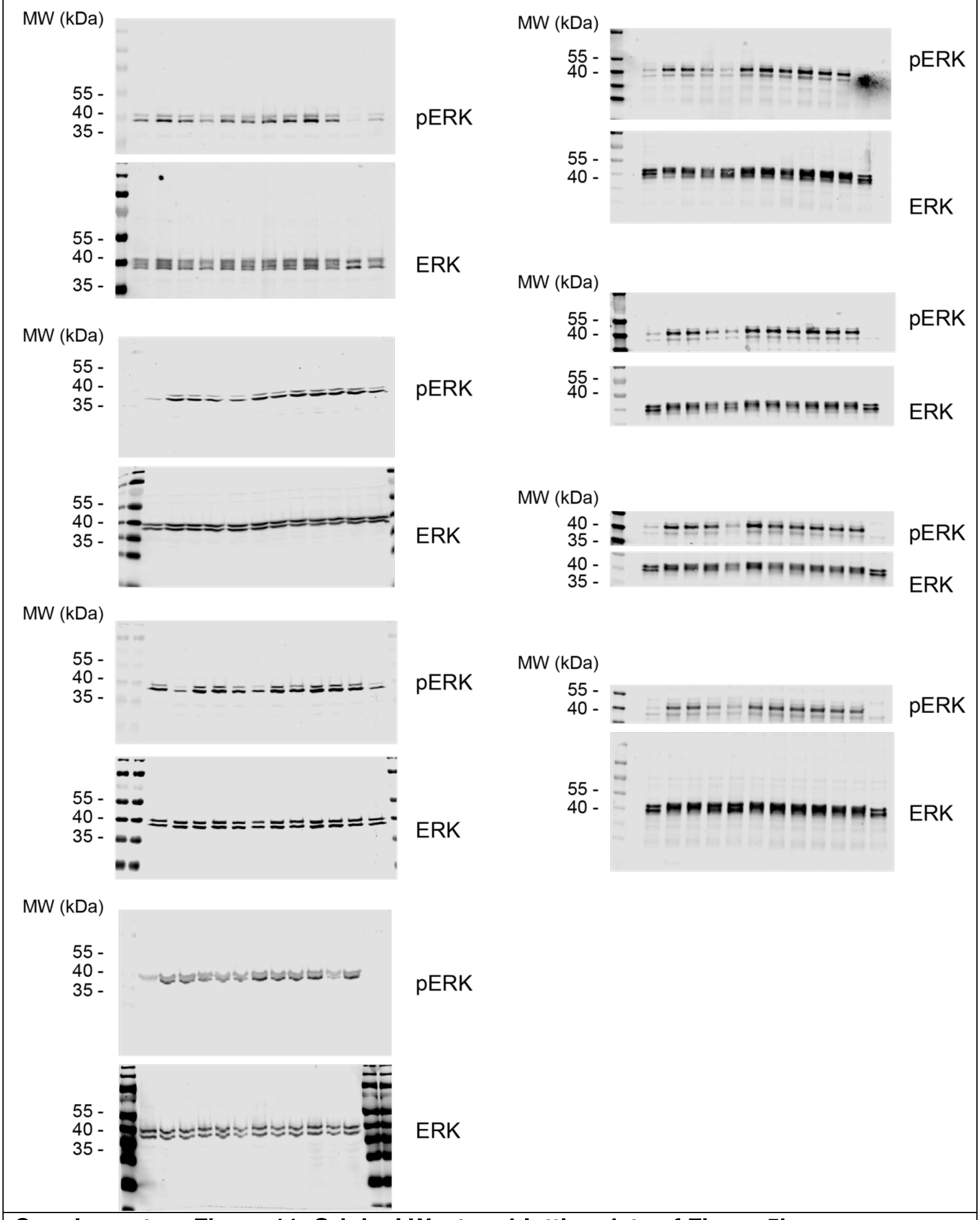

Supplementary Figure 11. Original Western blotting data of Figure 5b.

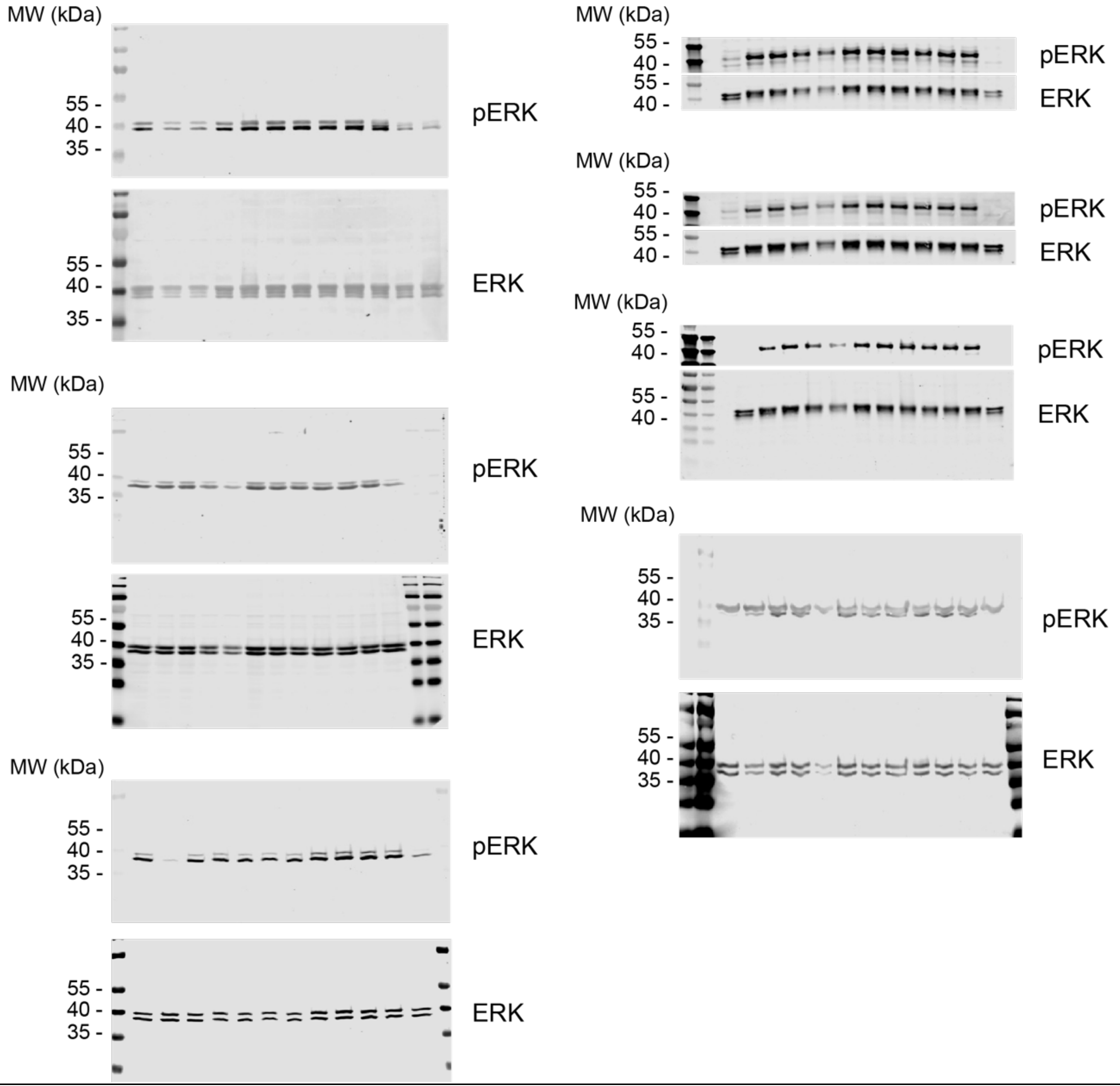

Supplementary Figure 12. Original Western blotting data of Figure 5c.

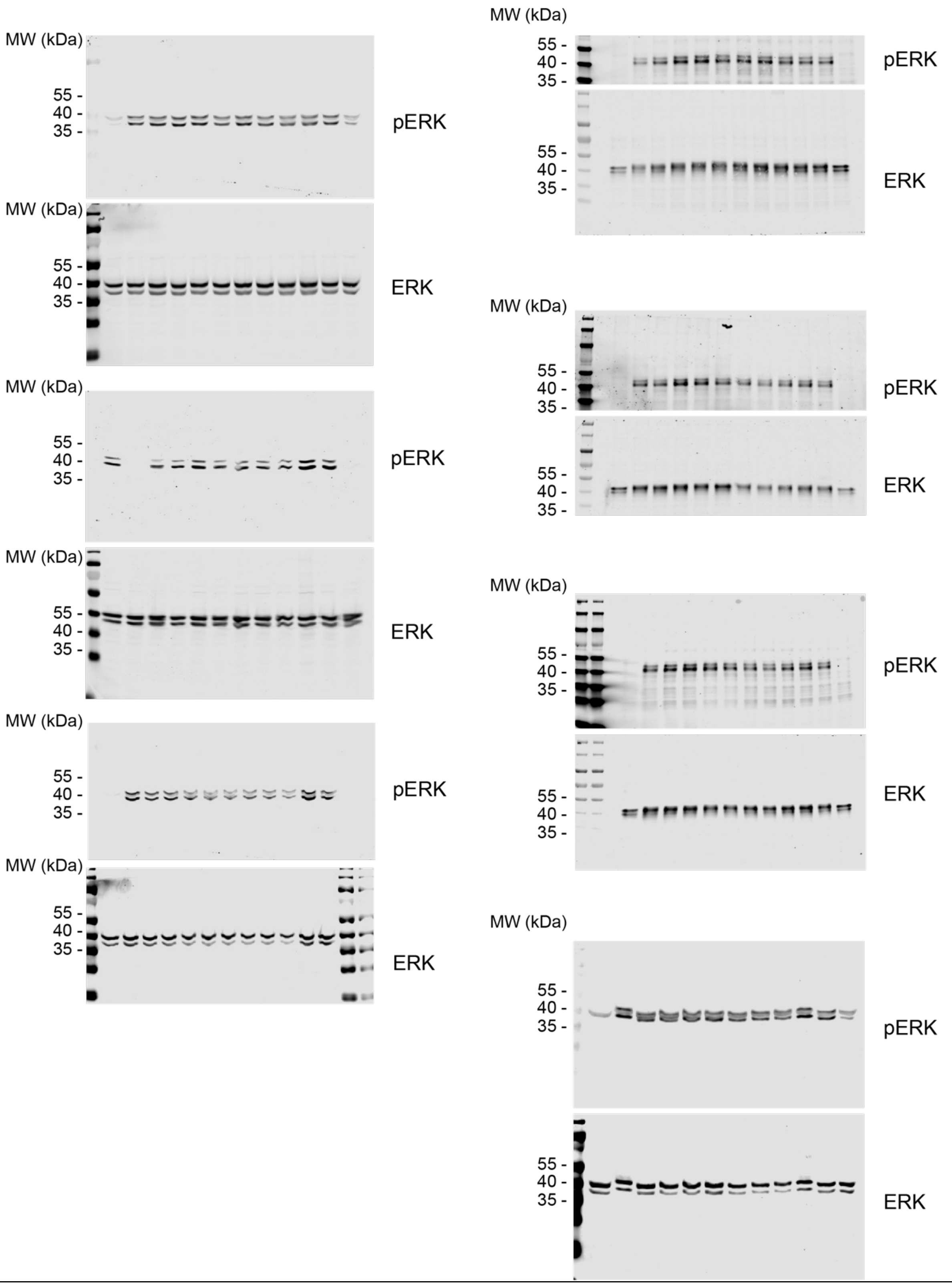

Supplementary Figure 13. Original Western blotting data of Figure 5d.

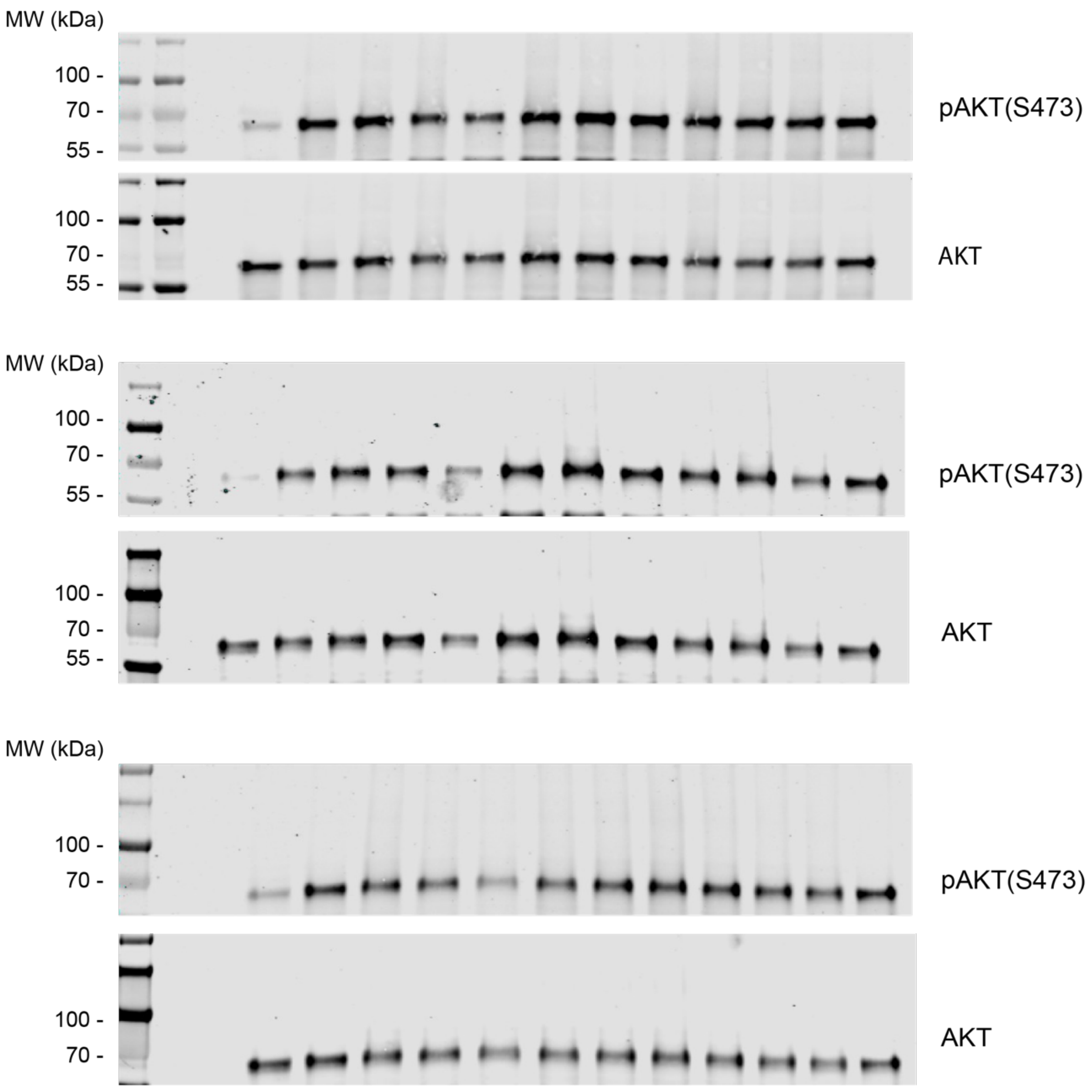

Supplementary Figure 14. Original Western blotting data of Figure 5e.

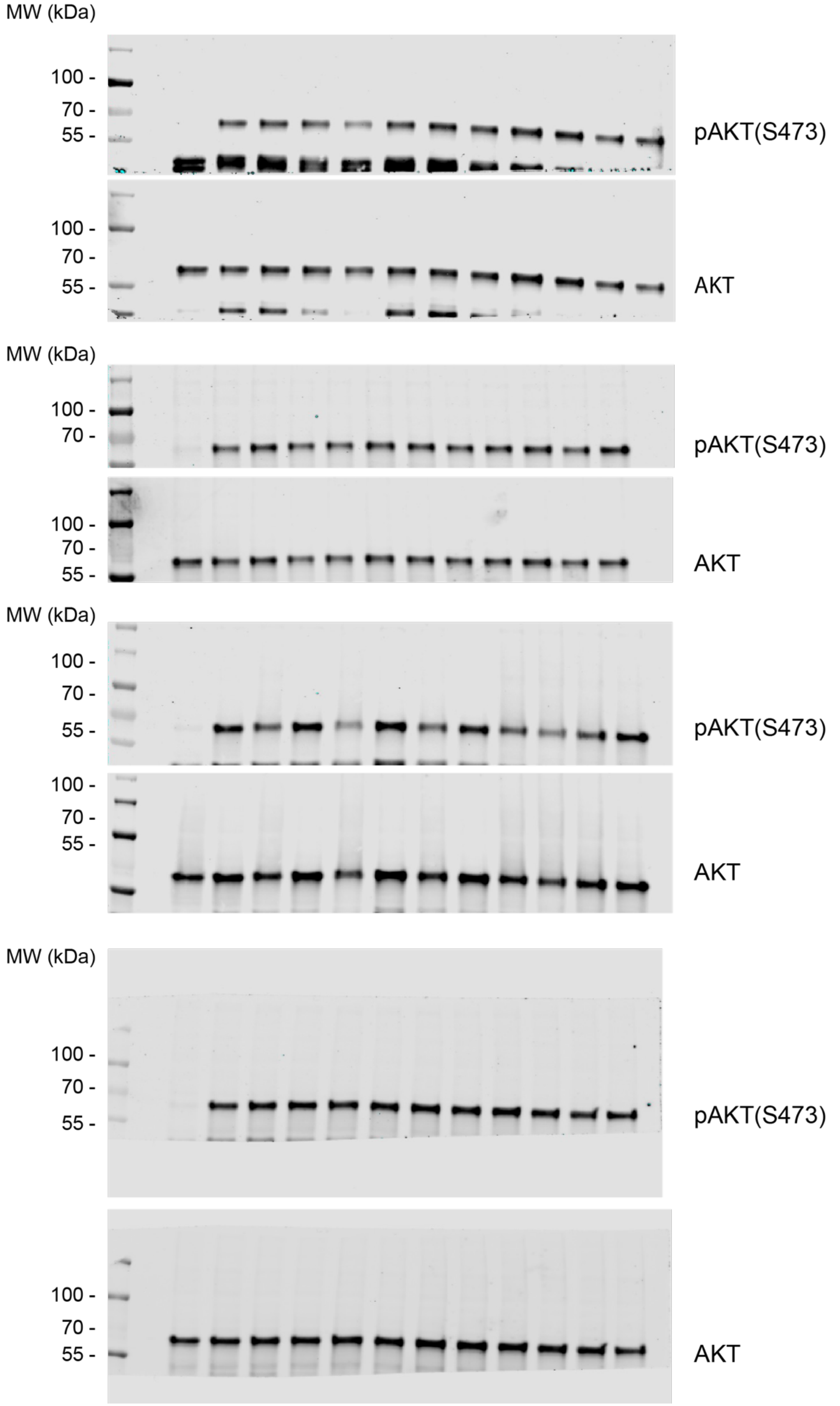

Supplementary Figure 15. Original Western blotting data of Figure 5f.

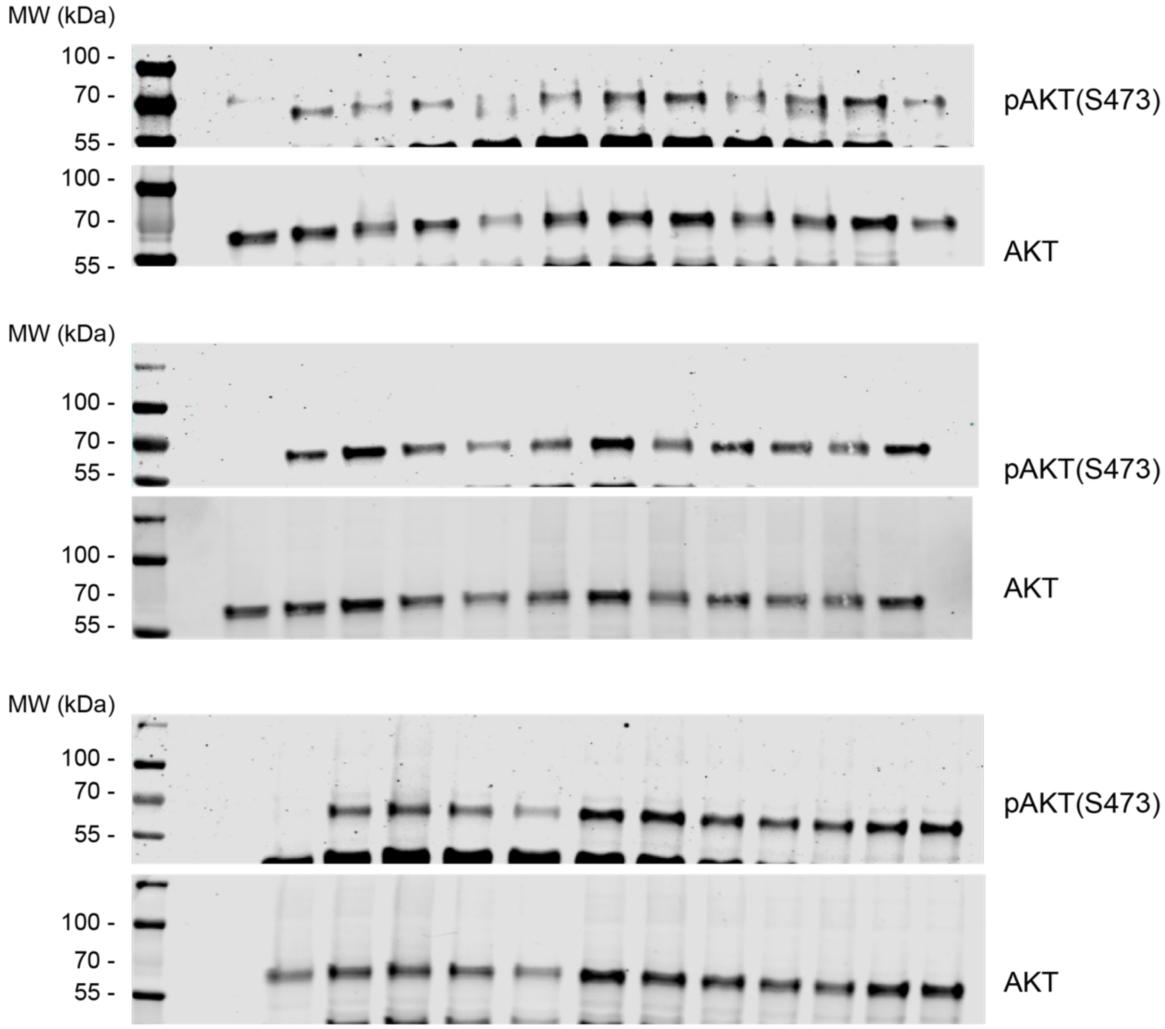

Supplementary Figure 16. Original Western blotting data of Figure 5g.

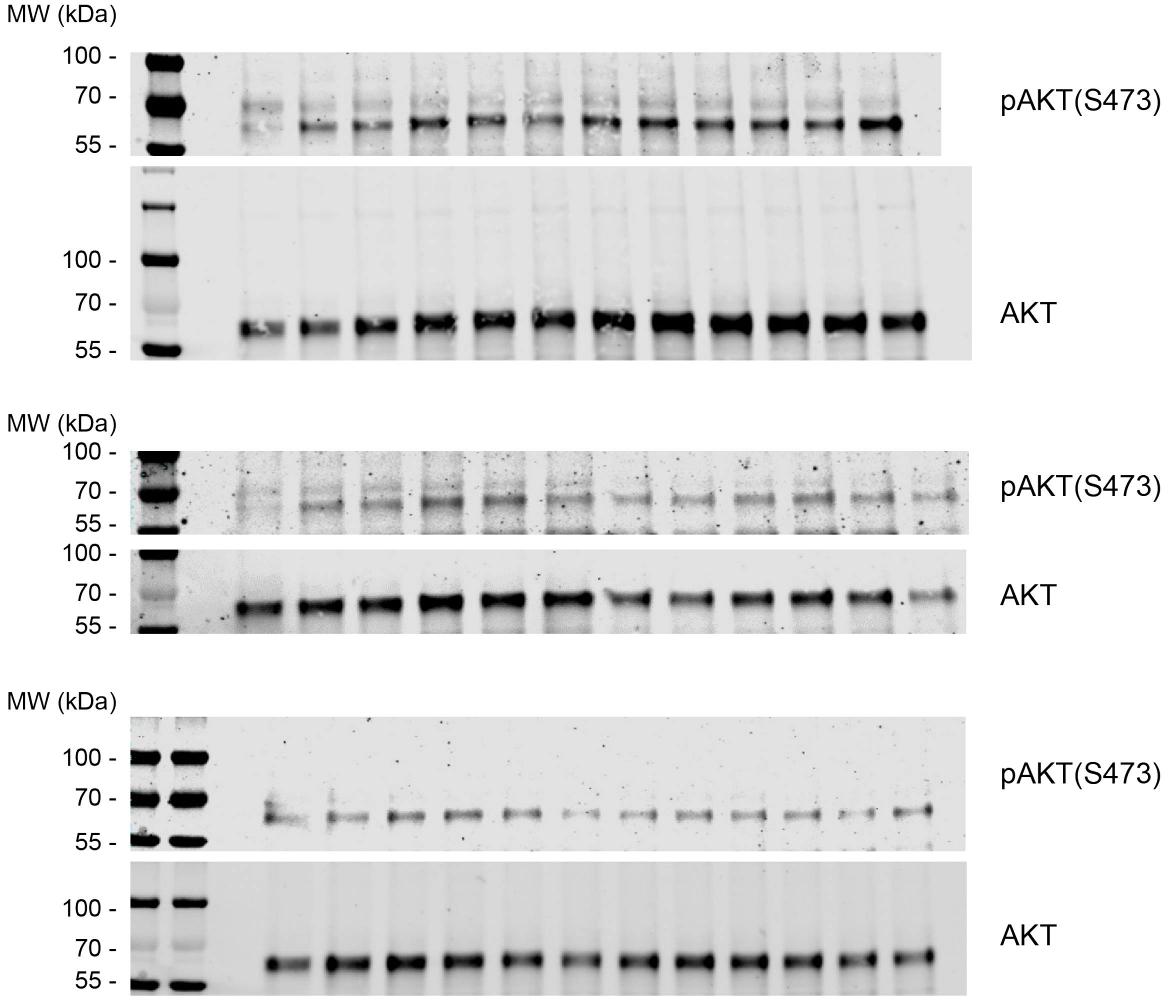

Supplementary Figure 17. Original Western blotting data of Figure 5h.

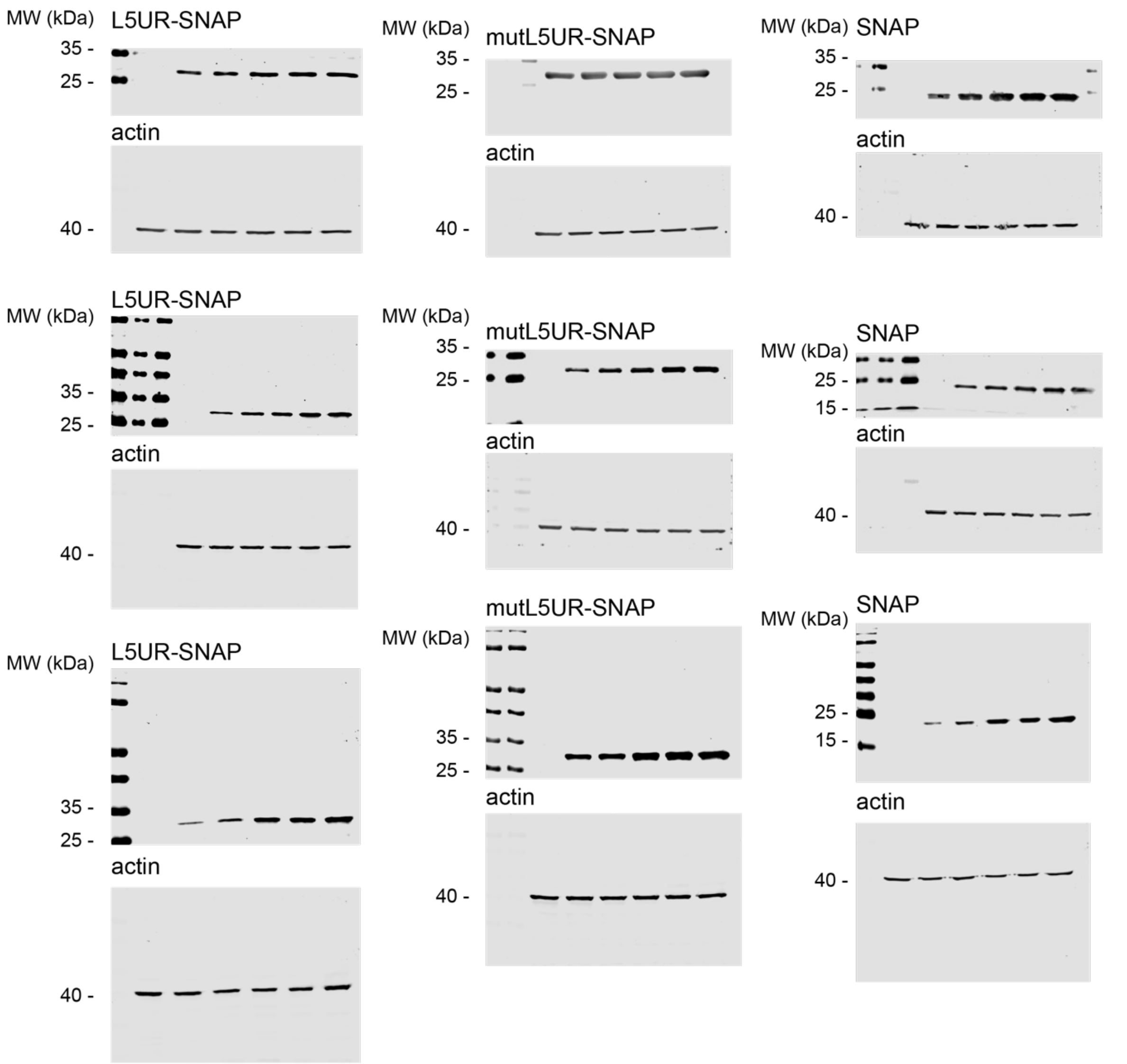

Supplementary Figure 18. Original Western blotting data of Figure S3a.

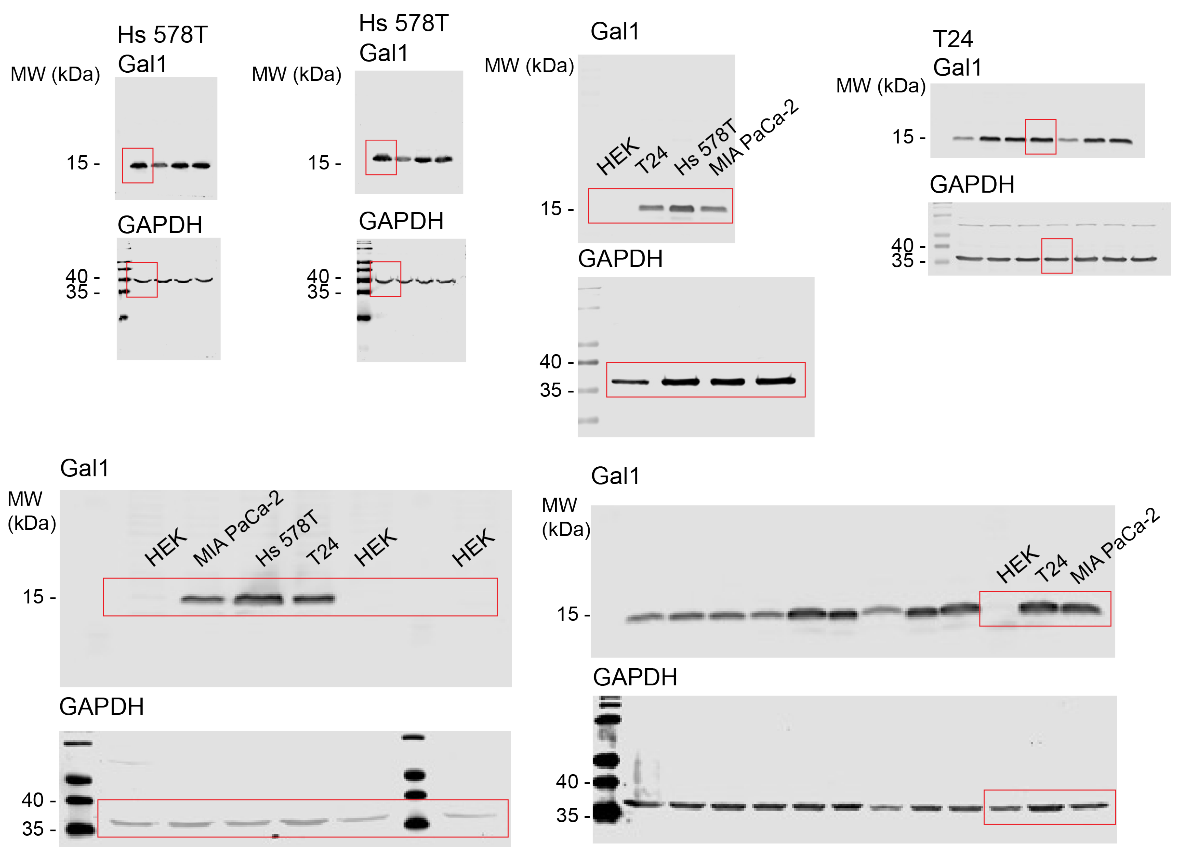

Supplementary Figure 19. Original Western blotting data of Figure S3d.

Supplementary Table 1: Materials and equipment employed in the study.

REAGENT or RESOURCE	SOURCE	IDENTIFIER
Antibodies		
mouse monoclonal anti-Galectin 1 (E2)	Santa Cruz Biotechnology	sc-166619 RRID:AB_2136629
mouse monoclonal Lambda 5 (A-1), λ5	Santa Cruz Biotechnology	sc-398932 RRID: N/A
rabbit polyclonal GST	Cell Signaling	2622S RRID: N/A
rabbit polyclonal anti-SNAP	New England Biolabs	P9310S RRID:AB_10631145
mouse monoclonal anti-B-Raf (F-7)	Santa Cruz Biotechnology	sc-5284 RRID:AB_626760
rabbit polyclonal anti-C-Raf (C-12)	Santa Cruz Biotechnology	sc-133 RRID:AB_632305
rabbit polyclonal anti-PI3K p110α	Cell Signaling	4255 RRID:AB_659888
mouse monoclonal anti-RASSF7 (C-6)	Santa Cruz Biotechnology	sc-374431 RRID:AB_10989731
rabbit polyclonal anti-RASSF9	Invitrogen	PA5-58878 RRID: N/A
rabbit polyclonal anti-ASPP2	Bethyl	A300-819A RRID:AB_597858
rabbit polyclonal anti-GAPDH	Sigma-Aldrich	G9545, RRID:AB_796208
mouse monoclonal anti-β-actin	Sigma-Aldrich	A5441 RRID:AB_476744
mouse monoclonal anti-phospho-p44/42 MAPK (Erk1/2) (Thr202/Tyr204) (E10)	Cell Signaling Technology	9106 RRID:AB_331768
rabbit polyclonal anti 44/42 MAPK (Erk1/2)	Cell Signaling Technology	9102 RRID:AB_330744
rabbit monoclonal anti-phospho-AKT(S473) (D9E)	Bioke	4060S RRID: N/A
mouse monoclonal anti-AKT(pan) (40D4)	Bioke	2920S RRID: N/A
IRDye 680LT Goat anti-Mouse IgG1-Specific Secondary Antibody	Li-Cor Biosciences	926-68052 RRID:AB_2783644
IRDye 800CW Goat anti-Mouse IgG Secondary Antibody	Li-Cor Biosciences	926-32210 RRID:AB_621842
IRDye 680RD Goat anti-Rabbit IgG Secondary Antibody	Li-Cor Biosciences	926-68071, RRID:AB_10956166
IRDye 800CW Goat anti-Rabbit IgG Secondary Antibody	LI-Cor Biosciences	926-32212, RRID:AB_621847
Bacterial and virus strains		
E. coli DH10B	New England Biolabs	C3019I
E. coli BL21 Star (DE3)pLysS	Thermo Fisher Scientific	C602003
Biological samples		
N/A	N/A	N/A
Chemicals, peptides, and recombinant proteins		
Fluorescein-isothiocyanate labelled L5UR	Pepmic Co., China	N/A
L5UR	Pepmic Co., China	N/A
mutL5UR	Pepmic Co., China	N/A

L5URcore	Pepmic Co., China	N/A
Biotinylated L5UR	This paper	N/A
TAT-L5URcore	This paper	N/A
TAT-mutL5URcore	This paper	N/A
TAT	This paper	N/A
Eu-L5URcore	This paper	N/A
Benzethonium chloride	Sigma-Aldrich	53751-50G; CAS121-54-0
Trametinib	MedChem Express	SC-364639; CAS871700-17-3
Pierce Protease Inhibitor Mini Tablets, EDTA-free	Thermo Scientific	#A32955
PhosSTOP	Roche	04 906 837 001
Critical commercial assays		
Gateway LR Clonase II enzyme mix	Thermo Fisher Scientific	11791020
jetPRIME transfection reagent	Polyplus	101000046
Coelenterazine 400a; 2,8-Dibenzyl-6-phenyl-imidazo[1,2a]pyrazin-3-(7H)-one; DeepBlueC	Gold Biotechnology	C-320-1
Coelenterazine h	Sanbio bv	16894-1
alamarBlue cell viability reagent	Thermo Fisher Scientific	DAL1100
SNAP-capture magnetic beads	New England Biolabs	S9145S
Experimental models: Cell lines		
Human cell line, HEK293-EBNA (HEK)	Prof. Florian M. Wurm, EPFL	RRID:CVCL_6974
Human cell line, MIA PaCa-2	ATCC	CRM-CRL-1420, RRID:CVCL_0428
Human cell line, Hs 578T	DSMZ	ACC 781, RRID:CVCL_0332
Human cell line, T24	DSMZ	ACC 376, RRID:CVCL_0554
BHK-21	DSMZ	CCL-10, RRID:CVCL_1914
Experimental models: Organisms/strains		
N/A		
Oligonucleotides		
N/A		
Recombinant DNA		
C413-E36_CMV promoter	3	Addgene, #162927
C453-E04_CMV promoter	3	Addgene, #162973
pDest-305	3	Addgene, #161895
pDest-312	3	Addgene, #161897
pDest-527		Addgene, #11518
C231-E13_RLuc8-stop	3	Addgene, FNL Combinatorial Cloning Platform, kit #1000000211
C511-E03_RLuc8-no stop	3	Addgene, FNL Combinatorial Cloning Platform, kit #1000000211
pDONR235-GFP2_stop	4	N/A
pDONR257-GFP2_no stop	4	N/A

Hs. K-Ras4B G12V (mutated P01116-2)	RAS mutant collection V2.0, RAS-Initiative	Addgene, #83132
Hs. H-Ras G12V (mutated P01112-1)	RAS mutant collection V2.0, RAS-Initiative	Addgene, #83184
Hs. ARAF (P10398)	RAS mutant collection V2.0, RAS-Initiative	Addgene, #70293
Hs. BRAF (P15056)	RAS mutant collection V2.0, RAS-Initiative	Addgene, #70299
Hs. RAF1 (P04049)	RAS mutant collection V2.0, RAS-Initiative	Addgene, #70497
pDONR221-hGal1 (P09382)	This paper	N/A
pDONR221-hNGal1 (mutated P09382)	This paper	N/A
pDONR221-C-RBD (P04049)	GeneCust (Boynes, France)	N/A
pDONR221-B-RBD (aa 155-227 of P15056)	GeneCust (Boynes, France)	N/A
pDest305-CMV-GFP2- K-Ras4BG12V (mutated P01116-2)	4	N/A
pDest305-CMV-RLuc8- K-Ras4BG12V (mutated P01116-2)	4	N/A
pDest305-CMV-GFP2- H-RasG12V (mutated P01112-1)	4	N/A
pDest305-CMV-RLuc8- H-RasG12V (mutated P01112-1)	4	N/A
pDest305-CMV-hGal1 (P09382)	This paper	N/A
pDest305-CMV-RLuc8-Gal1 (P09382)	This paper	N/A
pDest305-CMV-GFP2-Gal1 (P09382)	This paper	N/A
pDest305-CMV-RLuc8-N-hGal1 (mutated P09382)	This paper	N/A
pDest305-CMV-GFP2-N-hGal1 (mutated P09382)	This paper	N/A
pEF-A-RBD-GFP2 (aa 19-91 of P10398)	This paper	N/A
pEF-B-RBD-GFP2 (aa 155-227 of P15056)	This paper	N/A
pEF-C-RBD-GFP2 (aa 56-131 of P04049)	This paper	N/A
pClontech-C-L5UR (P15814-1)	This paper	N/A
pEF-L5UR-SNAP (aa 38-89 of P15814-1)	GeneCust (Boynes, France)	N/A
pEF-mutL5UR-SNAP (mutated aa 38-89 of P15814-1)	GeneCust (Boynes, France)	N/A
pEF-SNAP	GeneCust (Boynes, France)	N/A
pDest305-CMV-GFP2-B-Raf (P15056)	This paper	N/A
pDest305-CMV-GFP2-C-Raf (P04049)	This paper	N/A
pDest305-CMV-GFP2-A-Raf (P10398)	This paper	N/A
pEF-A-RBD-D75A-GFP2 (mutated aa 19-91 of P10398)	This paper	N/A
pEF-B-RBD-D211,213A-GFP2 (mutated aa 155-227 of P15056)	This paper	N/A
mGFP-rtGal1 (P11762)	1	N/A
mRFP-C-RBD (aa 56-131 of P04049)	5	N/A

mGFP-H-RasG12V (mutated P01112-1)	6	N/A
mCherry-H-RasG12V (mutated P01112-1)	7	N/A
mRFP-C-RBD-D117A (mutated, aa 56-131 of P04049)	1	N/A
pcDNA3-rtGal1 (P11762)	8	N/A
pcDNA3-N-rtGal-1 (mutated P11762)	1	N/A
pcDNA-Hygro-Anginex	9, 10	N/A
pDest527-His-hGal1 (P09382)	This paper	N/A
pGEX4T2-B-RBD (aa 155-227 of P15056)	This paper	N/A
pGEX2T-C-RBD (aa 50-134 of P04049)	This paper	N/A
pGEX4T2	Addgene	27458101
pcDNA3.1(-)	ThermoFisher Scientific	V79520
pDest305-CMV-mNeonGreen- H-RasG12V (mutated P01112-1)	This paper	N/A
pDest305-CMV-NanoLuc- H-RasG12V (mutated P01112-1)	This paper	N/A
pcDNA3-RLucF1-BRAF-RLucF2	11	N/A
Software and algorithms		
BREEZE pipeline	12	https://breeze.fimm.fi/
PyMol	The PyMOL Molecular Graphics System	https://pymol.org/2/
GraphPad Prism v9.5.1	GraphPad by Dotmatics,	https://www.graphpad.com/
Other		
CLARIOstar Plus Microplate Reader	BMG LABTECH	https://www.bmglabtech.com/en/clariostar-plus/
Odyssey CLx Infrared Imaging System	LI-COR Biosciences	https://www.licor.com/bio/odyssey-clx/
ÄKTA pure chromatography system	Cytiva	https://www.cytivalifesciences.com/en/us/shop/chromatography/chromatography-systems/akta-pure-p-05844
Elmasonic S 40 H	Elma	https://www.elma-ultrasonic.com/
Tecan Spark multimode microplate reader	Tecan Austria GmbH	https://lifesciences.tecan.com/multimode-plate-reader
Electron microscope	JEOL	JEOL JEM-1400

Inverted microscope AXIO Observer D1	Zeiss	https://www.zeiss.com/microscopy/en/products/light-microscopes/widefield-microscopes/axio-observer-for-life-science-research.html#features
Lambert Instruments FLIM Attachment (LIFA)	Lambert Instruments	https://www.lambertinstruments.com/lifa#lifa-introduction
LM10 Microfluidizer Processor	(Microfluidics, USA)	https://www.microfluidics-mpt.com/microfluidizers/lm10

Supplementary Table 2: Sequences, N-terminal modifications, calculated and found m/z values of synthesized peptides. All the peptides bear a C-terminal amide. (Ac: Acetylated)

**Peptide**	**Sequence**	**N-term.**	**Purity / %**	**m/z calc.**	**m/z found**
L5UR	LLRPTAASQSRALGPGAPGGSSRSSLRSRWGRFLLQRGSWTGPRCWPRGFQS	Ac<br>Biotin-PEG5	90<br>>95	949.8<br>1234.6	950.2 $[M+6H]^{+6}$<br>1235.4 $[M+5H]^{+5}$
L5URcore	SRSSLRSRWGRFLLQRGSWTGPR	Ac	>95	929.8	930.2 $[M+3H]^{+3}$
L5URcore-nK	KSRSSLRSRWGRFLLQRGSWTGPR	Ac	>95	1458.1	1458.2 $[M+2H]^{+2}$
mutL5UR core	SRSSDEEEGGRESLQRGSWTGPR	Ac	>95	868.7	869.0 $[M+3H]^{+3}$
TAT	GRKKRRQRRRPQ	Ac	>95	555.0	555.1 $[M+3H]^{+3}$
TAT-PEG2-L5URcore	GRKKRRQRRRPQ-PEG2-SRSSLRSRWGRFLLQRGSWTGPR	Ac	>95	648.8	649.1 $[M+7H]^{+7}$
TAT-PEG2-mutL5UR core	GRKKRRQRRRPQ-PEG2-SRSSDEEEGGRESLQRGSWTGPR	Ac	>95	1088.8	1089.3 $[M+4H]^{+4}$

Supplementary Information References

1. Blazevits O*, et al.* Galectin-1 dimers can scaffold Raf-effectors to increase H-ras nanoclustering. *Sci Rep* **6**, 24165 (2016).

2. Elantak L*, et al.* Structural basis for galectin-1-dependent pre-B cell receptor (pre-BCR) activation. *J Biol Chem* **287**, 44703-44713 (2012).

3. Wall VE, Garvey LA, Mehalko JL, Procter LV, Esposito D. Combinatorial assembly of clone libraries using site-specific recombination. *Methods Mol Biol* **1116**, 193-208 (2014).

4. Okutachi S*, et al.* A Covalent Calmodulin Inhibitor as a Tool to Study Cellular Mechanisms of K-Ras-Driven Stemness. *Front Cell Dev Biol* **9**, 665673 (2021).

5. Abankwa D, Gorfe AA, Inder K, Hancock JF. Ras membrane orientation and nanodomain localization generate isoform diversity. *Proc Natl Acad Sci U S A* **107**, 1130-1135 (2010).

6. Abankwa D*, et al.* A novel switch region regulates H-ras membrane orientation and signal output. *EMBO J* **27**, 727-735 (2008).

7. Solman M*, et al.* Specific cancer-associated mutations in the switch III region of Ras increase tumorigenicity by nanocluster augmentation. *Elife* **4**, e08905 (2015).

8. Paz A, Haklai R, Elad-Sfadia G, Ballan E, Kloog Y. Galectin-1 binds oncogenic H-Ras to mediate Ras membrane anchorage and cell transformation. *Oncogene* **20**, 7486-7493 (2001).

9. Thijssen VL*, et al.* Galectin-1 is essential in tumor angiogenesis and is a target for antiangiogenesis therapy. *Proc Natl Acad Sci U S A* **103**, 15975-15980 (2006).

10. Brandwijk RJ, Dings RP, van der Linden E, Mayo KH, Thijssen VL, Griffioen AW. Anti-angiogenesis and anti-tumor activity of recombinant anginex. *Biochem Biophys Res Commun* **349**, 1073-1078 (2006).

11. Rock R*, et al.* BRAF inhibitors promote intermediate BRAF(V600E) conformations and binary interactions with activated RAS. *Sci Adv* **5**, eaav8463 (2019).

12. Potdar S*, et al.* Breeze 2.0: an interactive web-tool for visual analysis and comparison of drug response data. *Nucleic Acids Res*, (2023).
