# Supplementary material for: Identification of an H-Ras nanocluster disrupting peptide
Source: Commun Biol. 2024 Jul 9;7:837. doi: 10.1038/s42003-024-06523-9 (PMC11233548; doi:10.1038/s42003-024-06523-9)
Supplement: Supplementary file 5 — Reporting summary [file 42003_2024_6523_MOESM5_ESM.pdf]

nature portfolio

Corresponding author(s): daniel.abankwa@uni.lu

Last updated by author(s): Jun 9, 2024

Reporting Summary

Nature Portfolio wishes to improve the reproducibility of the work that we publish. This form provides structure for consistency and transparency in reporting. For further information on Nature Portfolio policies, see our Editorial Policies and the Editorial Policy Checklist.

Statistics

For all statistical analyses, confirm that the following items are present in the figure legend, table legend, main text, or Methods section.

n/a	Confirmed	
☐	☒	The exact sample size (*n*) for each experimental group/condition, given as a discrete number and unit of measurement
☐	☒	A statement on whether measurements were taken from distinct samples or whether the same sample was measured repeatedly
☐	☒	The statistical test(s) used AND whether they are one- or two-sided<br>*Only common tests should be described solely by name; describe more complex techniques in the Methods section.*
☒	☐	A description of all covariates tested
☒	☐	A description of any assumptions or corrections, such as tests of normality and adjustment for multiple comparisons
☐	☒	A full description of the statistical parameters including central tendency (e.g. means) or other basic estimates (e.g. regression coefficient) AND variation (e.g. standard deviation) or associated estimates of uncertainty (e.g. confidence intervals)
☐	☒	For null hypothesis testing, the test statistic (e.g. *F*, *t*, *r*) with confidence intervals, effect sizes, degrees of freedom and *P* value noted<br>*Give P values as exact values whenever suitable.*
☒	☐	For Bayesian analysis, information on the choice of priors and Markov chain Monte Carlo settings
☒	☐	For hierarchical and complex designs, identification of the appropriate level for tests and full reporting of outcomes
☒	☐	Estimates of effect sizes (e.g. Cohen's *d*, Pearson's *r*), indicating how they were calculated

Our web collection on statistics for biologists contains articles on many of the points above.

Software and code

Policy information about availability of computer code

Data collection	For BRET, FP and 2D data acquisition ClarioStar Software 5.21 R4 was used.<br>For FRET data collection the software Li-FLIM version 1.2.25 provided with the FLIM-FRET-setup as used.<br>EM nanocluster analysis data collection was made with the camera software from Gatan Inc. version 2.02.800.0.<br>QRET data collection the software SparkControl with the Tecan Spark 20M (Tecan Lifesciences, Männedorf, Switzerland) was used.
Data analysis	For BRET, FP, FRET, QRET, 2D data analysis Graphpad Prism v9.5.1. to v10.1.0 were used.<br>For Western blot quantification either Image Studio5.2 provided with Odyssey CLx Infrared Imaging System or Fiji (ImageJ 2.14.0) were used.<br>The statistical K-function analyses for both univariate and bivariate nanoclustering in EM nanocluster analysis employed custom algorithms written in Chipmunk Basic and macros routines in Excel. Both routines are available upon request.<br>Survival data analysis was performed in R version 4.2.1. using survival V3.4 and survminer V0.4 libraries.<br>2D proliferation data was analysed using the Breeze 2.0. pipeline (https://breeze.fimm.fi/)<br>Protein sequences were aligned using Clustal Omega (https://www.ebi.ac.uk/Tools/msa/clustalo/.<br>Protein structures figures and analysis were generated using PyMOL Molecular Graphics System (Version 2.5.1). All PDB files used for figures downloaded from the www.rcsb.org database.

For manuscripts utilizing custom algorithms or software that are central to the research but not yet described in published literature, software must be made available to editors and reviewers. We strongly encourage code deposition in a community repository (e.g. GitHub). See the Nature Portfolio guidelines for submitting code & software for further information.

Data

Policy information about availability of data

All manuscripts must include a data availability statement. This statement should provide the following information, where applicable:

- Accession codes, unique identifiers, or web links for publicly available datasets
- A description of any restrictions on data availability
- For clinical datasets or third party data, please ensure that the statement adheres to our policy

All relevant data supporting this study are available within the manuscript and supplementary data. Source data are provided with the manuscript. All unique/stable reagents generated in this study are available from the corresponding author with a completed materials transfer agreement. This study did not report standardized datatypes.

Human research participants

Policy information about studies involving human research participants and Sex and Gender in Research.

Reporting on sex and gender	N/A
Population characteristics	N/A
Recruitment	N/A
Ethics oversight	N/A

Note that full information on the approval of the study protocol must also be provided in the manuscript.

Field-specific reporting

Please select the one below that is the best fit for your research. If you are not sure, read the appropriate sections before making your selection.

☒ Life sciences ☐ Behavioural & social sciences ☐ Ecological, evolutionary & environmental sciences

For a reference copy of the document with all sections, see nature.com/documents/nr-reporting-summary-flat.pdf

Life sciences study design

All studies must disclose on these points even when the disclosure is negative.

Sample size	The sample size for the experiments was determined based on similar experiments from previous publications. No statistical method was used to predetermine sample size. In general, all the experiments were performed with at least two independent biological repeats to ensure reproducibility. The sample size (n) is provided in the figure legends.
Data exclusions	No data was excluded within shown datasets.
Replication	The number of repeats for the experiments is indicated in the figure panels. The type of repeats is indicated in the method section.
Randomization	To our best understanding no randomization was needed for the experiments conducted based on common scientific practices related to the field, hence no randomization was needed.
Blinding	Based on common scientific practices related to the field and similar experiments from previous publications, no blinding was performed. The study does not involve any comparisons between experimental groups.

Reporting for specific materials, systems and methods

We require information from authors about some types of materials, experimental systems and methods used in many studies. Here, indicate whether each material, system or method listed is relevant to your study. If you are not sure if a list item applies to your research, read the appropriate section before selecting a response.

Materials & experimental systems

n/a	Involved in the study
☐	☒ Antibodies
☐	☒ Eukaryotic cell lines
☒	☐ Palaeontology and archaeology
☒	☐ Animals and other organisms
☒	☐ Clinical data
☒	☐ Dual use research of concern

Methods

n/a	Involved in the study
☒	☐ ChIP-seq
☒	☐ Flow cytometry
☒	☐ MRI-based neuroimaging

Antibodies

Antibodies used

The following primary antibodies were used: mouse monoclonal anti-Galectin 1 (E2) (Santa Cruz Biotechnology sc-166619/B0811) at 1:2000; mouse monoclonal Lambda 5 (A-1) (Santa Cruz Biotechnology sc-398932) at 1:2000; rabbit polyclonal GST (Cell Signalling 2622S) at 1:10000; rabbit polyclonal anti-SNAP (NEB P9310S) at 1:5000; mouse monoclonal anti-B-Raf (F-7) (Santa Cruz Biotechnology sc-5284) at 1:200; rabbit polyclonal anti-C-Raf (C-12) (Santa Cruz Biotechnology sc-133) at 1:2000; rabbit polyclonal anti-PI3K p110α (Cell Signalling, #4255) at 1:1000, mouse monoclonal anti-RASSF7 (C-6) (Santa Cruz Biotechnology sc-374431) at 1:500, rabbit polyclonal anti-RASSF9 (Invitrogen PA5-58878) at 1:1000, rabbit polyclonal anti-ASPP2 (Bethyl A300-819A) at 1:1000, rabbit polyclonal anti-GAPDH (Sigma-Aldrich G9545-200UL) at 1:10000;mouse monoclonal anti-B-actin (Sigma-Aldrich A5441-0.2ML / 0000126949) at 1:10000; mouse monoclonal anti-phospho-p44/42 MAPK (ERK1/2) (Cell Signalling, #9106) at 1:2000; rabbit polyclonal anti-p44/42 MAPK (Erk1/2) (Cell Signalling, #9102) at 1:1000; rabbit monoclonal anti-phospho-AKT(S473) (D9E) (Bioke, #4060S) at 1:1000 and mouse monoclonal anti-AKT(pan) (40D4) (Bioke, #2920S) at 1:1000.

The following secondary antibodies were used:
IRDye 680LT Donkey anti-Mouse IgG-Specific Secondary Antibody (Li-Cor, #926-68022) at 1:10000, IRDye 800CW Goat anti-Rabbit IgG Secondary Antibody (Li-Cor, #926-32211) at 1:10000, IRDye 680RD Goat anti-Rabbit IgG Secondary Antibody (Li-Cor, #926-68071) at 1:10000, IRDye 800CW Donkey anti-Mouse IgG Secondary Antibody (Li-Cor, #926-32212) at 1:10000.

Validation

All antibodies are validated for species and application by the manufacturer and validation can be found on manufacturers' websites using antibody references provided in the section "Antibodies used": mouse monoclonal anti-Galectin 1 (E2) (Santa Cruz Biotechnology sc-166619/B0811) at 1:2,000, https://www.scbt.com/de/p/galectin-1-antibody-e-2#citations; mouse monoclonal Lambda 5 (A-1) (Santa Cruz Biotechnology sc-398932) at 1:2,000, https://www.scbt.com/de/p/lambda-5-antibody-a-1; rabbit polyclonal GST (Cell Signalling 2622S) at 1:10,000, https://www.cellsignal.com/products/primary-antibodies/gst-tag-antibody/2622; rabbit polyclonal anti-SNAP (NEB P9310S) at 1:5,000, https://www.neb.com/en/products/p9310-anti-snap-tag-antibody-polyclonal#Citations%20&%20Technical%20Literature; mouse monoclonal anti-B-Raf (F-7) (Santa Cruz Biotechnology sc-5284) at 1:200, https://www.scbt.com/de/p/raf-b-antibody-f-7; rabbit polyclonal anti-C-Raf (C-12) (Santa Cruz Biotechnology sc-133) at 1:2000, https://www.scbt.com/de/p/raf-1-antibody-c-12; rabbit polyclonal anti-PI3K p110α (Cell Signalling, #4255) at 1:1000, https://www.cellsignal.com/products/primary-antibodies/pi3-kinase-p110a-antibody/4255; mouse monoclonal anti-RASSF7 (C-6) (Santa Cruz Biotechnology sc-374431) at 1:500, https://www.scbt.com/de/p/rassf7-antibody-c-6; rabbit polyclonal anti-RASSF9 (Invitrogen PA5-58878) at 1:1000, https://www.thermofisher.com/antibody/product/RASSF9-Antibody-Polyclonal/PA5-58878; rabbit polyclonal anti-ASPP2 (Bethyl A300-819A) at 1:1000, https://www.sanbio.nl/a300-819a-t-1; rabbit polyclonal anti-GAPDH (Sigma-Aldrich G9545-200UL) at 1:10,000, https://www.sigmaaldrich.com/LU/fr/product/sigma/g9545; mouse monoclonal anti-B-actin (Sigma-Aldrich A5441-0.2ML / 0000126949) at 1:10000, https://www.sigmaaldrich.com/LU/fr/product/sigma/a5441; mouse monoclonal anti-phospho-p44/42 MAPK (ERK1/2) (Cell Signalling, #9106) at 1:2000, https://www.cellsignal.com/products/primary-antibodies/phospho-p44-42-mapk-erk1-2-thr202-tyr204-e10-mouse-mab/9106 and rabbit polyclonal anti-p44/42 MAPK (Erk1/2) (Cell Signalling, #9102) at 1:1000, https://www.cellsignal.com/products/primary-antibodies/p44-42-mapk-erk1-2-antibody/9102; rabbit monoclonal anti-phospho-AKT(S473) (D9E) (Bioke, #4060S) at 1:1000, https://www.cellsignal.com/product/productDetail.jsp?productId=4060&country=LU; and mouse monoclonal anti-AKT(pan) (40D4) (Bioke, #2920S) at 1:1000, https://www.cellsignal.com/product/productDetail.jsp?productId=2920&country=LU.

The following secondary antibodies were used:
IRDye 680LT Donkey anti-Mouse IgG-Specific Secondary Antibody (Li-Cor, #926-68022) at 1:10000, https://www.licor.com/bio/reagents/irdye-680lt-donkey-anti-mouse-igg-secondary-antibody; IRDye 800CW Goat anti-Rabbit IgG Secondary Antibody (Li-Cor, #926-32211) at 1:10000, https://www.licor.com/bio/reagents/irdye-800cw-goat-anti-rabbit-igg-secondary-antibody; IRDye 680RD Goat anti-Rabbit IgG Secondary Antibody (Li-Cor, #926-68071) at 1:10000, https://www.licor.com/bio/reagents/irdye-680rd-goat-anti-rabbit-igg-secondary-antibody and IRDye 800CW Donkey anti-Mouse IgG Secondary Antibody (Li-Cor, #926-32212) at 1:10000, https://www.licor.com/bio/reagents/irdye-800CW-donkey-anti-mouse-igg-secondary-antibody.

Eukaryotic cell lines

Policy information about cell lines and Sex and Gender in Research

Cell line source(s)

The following cell lines were used: HEK293-EBNA (HEK, RRID:CVCL_6974), MIA PaCa-2 (CRM-CRL-1420, RRID:CVCL_0428), Hs 578T (ACC 781, RRID:CVCL_0332), T24 (ACC 376, RRID:CVCL_0554) and BHK-21 (CCL-10, RRID:CVCL_1914). HEK cells were a gift from Prof. Florian M. Wurm, EPFL. MIA PaCa-2 cells were purchased from ATCC. T24, Hs 578T and BHK-21 cells were from DSMZ, Braunschweig, Germany.

Authentication	The HEK293-ebna cells were authenticated by Eurofins Genomics, Ebersberg, Germany. All cell lines received from a company were not authenticated after reception.
Mycoplasma contamination	Cells were routinely tested for mycoplasma contamination using MycoAlert Plus mycoplasma Detection kit (Lonza, #LT07-710).
Commonly misidentified lines (See ICLAC register)	No commonly misidentified cell lines were used in this study.
